# Graph Analysis of Functional Brain Networks in Patients with Mild Traumatic Brain Injury

**DOI:** 10.1371/journal.pone.0171031

**Published:** 2017-01-27

**Authors:** Harm J. van der Horn, Edith J. Liemburg, Myrthe E. Scheenen, Myrthe E. de Koning, Jacoba M. Spikman, Joukje van der Naalt

**Affiliations:** 1 Department of Neurology of the University of Groningen, University Medical Center Groningen, Groningen, The Netherlands; 2 BCN NeuroImaging Center and Department of Neuroscience of the University of Groningen, University Medical Center Groningen, Groningen, The Netherlands; 3 Department of Neuropsychology of the University of Groningen, University Medical Center Groningen, Groningen, The Netherlands; University of Cambridge, UNITED KINGDOM

## Abstract

Mild traumatic brain injury (mTBI) is one of the most common neurological disorders worldwide. Posttraumatic complaints are frequently reported, interfering with outcome. However, a consistent neural substrate has not yet been found. We used graph analysis to further unravel the complex interactions between functional brain networks, complaints, anxiety and depression in the sub-acute stage after mTBI. This study included 54 patients with uncomplicated mTBI and 20 matched healthy controls. Posttraumatic complaints, anxiety and depression were measured at two weeks post-injury. Patients were selected based on presence (n = 34) or absence (n = 20) of complaints. Resting-state fMRI scans were made approximately four weeks post-injury. High order independent component analysis resulted in 89 neural components that were included in subsequent graph analyses. No differences in graph measures were found between patients with mTBI and healthy controls. Regarding the two patient subgroups, degree, strength, local efficiency and eigenvector centrality of the bilateral posterior cingulate/precuneus and bilateral parahippocampal gyrus were higher, and eigenvector centrality of the frontal pole/ bilateral middle & superior frontal gyrus was lower in patients with complaints compared to patients without complaints. In patients with mTBI, higher degree, strength and eigenvector centrality of default mode network components were related to higher depression scores, and higher degree and eigenvector centrality of executive network components were related to lower depression scores. In patients without complaints, one extra module was found compared to patients with complaints and healthy controls, consisting of the cingulate areas. In conclusion, this research extends the knowledge of functional network connectivity after mTBI. Specifically, our results suggest that an imbalance in the function of the default mode- and executive network plays a central role in the interaction between emotion regulation and the persistence of posttraumatic complaints.

## Introduction

Cognitive and affective complaints reported by patients with mild traumatic brain injury (mTBI) still puzzle clinicians and scientists worldwide [1–4]. In the majority of patients with mTBI, these complaints are present without any impairments objectified with neuropsychological assessment [5–7] and/or abnormalities visible on computed tomography (CT) or conventional structural magnetic resonance imaging (MRI) scans [8–11]. A growing number of functional MRI (fMRI) studies suggest that (persistent) posttraumatic complaints after mTBI are associated with alterations in functional brain networks, especially with regard to the interaction between frontal/parietal networks, such as the default mode network, executive network and salience network [12–16]. This is not surprising, since these networks converge on prefrontal midline areas, which are vulnerable to traumatic brain injury [17–19], and because these networks and regions are important for emotion regulation [20,21]. However, much uncertainty still exists about the exact role of functional network dynamics in the pathophysiological mechanisms underlying sequelae of mTBI.

To gain a comprehensive understanding of the specific features of network dysfunction that are involved in cognitive and emotional consequences of mTBI, it might be useful to use more sophisticated measures of network function [22]. A computational method that is increasingly being used to study functional brain networks in various neurological conditions is graph analysis, which is derived from graph theory [23–26]. The basis of graph analysis of resting-state fMRI is functional connectivity, which is defined as the statistical correlation between blood oxygen level dependent (BOLD) responses of separate regions (i.e. nodes (*N*)) throughout the brain [23]. An *N* x *N* functional connectivity matrix is constructed, and subsequently graph analysis is applied to this matrix in order to gain knowledge about the connections and hierarchy of nodes as well as about local and global architecture throughout the network. There are several advantages of graph analysis over traditional within- and between-network functional connectivity analysis techniques such as seed-based analysis, region of interest (ROI) analysis, and (low order) independent component analysis (ICA) [24,27,28]. First, with graph analysis it is possible to calculate measures of global network function, such as global efficiency or average path length. Second, graph analysis allows us to capture complex local interactions, because it not only provides information about the bidirectional relationship between two nodes or components, but also about neighboring nodes, neighborhoods of a node and subnetworks. Third, the importance (‘hub status’) of individual nodes for the network can be determined [28]. Thus, this analysis technique covers a wider range of network aspects compared to traditional methods.

Until now, only a small number of studies have used graph analysis to study functional networks in patients with mTBI [29,30]. Among other things, these studies have shown an association between lower local efficiency of the prefrontal cortex and basal ganglia, and a higher severity of posttraumatic complaints and stress. In the present study, exploratory graph analysis was performed in a relatively large sample of patients with uncomplicated mTBI. Especially, differences between patients with and without complaints, and associations with anxiety and depression were examined. It was hypothesized that local graph measures of prefrontal midline areas would be affected because of their vulnerability to TBI and their role in network dynamics involved in cognition and emotion regulation. High order ICA was used to define network nodes [31]. This data-driven method does not require any a priori hypotheses about specific features of brain networks, and has served well in the examination of network dysfunction in a wide variety of neurological and psychiatric diseases, including TBI [14,32]. Furthermore, ICA has proven useful in defining nodes for graph analysis studies of patients with more severe TBI [33,34]. In the current analyses, a high order ICA model (i.e. with a high number of components) was applied, which may be more accurate in functional segmentation of the brain [35,36], and may result in better delineation of disease related functional connectivity alterations than lower order ICA [37].

## Methods

### Participants

This study elaborates on previously published work of our research group using the same patient sample [13]. For information on recruitment of participants and behavioural data analyses we refer the reader to the method section of that paper. Study approval was obtained from the local Medical Ethics Committee of the University Medical Center Groningen, the Netherlands, and all participants provided written informed consent after the study and procedure had been fully explained. All study procedures were carried out according to the declaration of Helsinki.

Fifty-four patients (18–65 years of age) with mTBI (Glasgow coma scale 13–15 and/or loss of consciousness ≤ 30 minutes [38]) were enrolled. Patients were selected based on the number of self-reported complaints on a head injury symptoms checklist (HISC) [4] administered at two weeks post-injury (pre-injury scores subtracted from post-injury scores): a group with posttraumatic complaints (PTC-present; n = 34) defined as ≥3 complaints and a group without complaints (PTC-absent; n = 20) defined as <3 complaints [39–42]. In addition, a healthy control group (n = 20) was included that was age-, sex, education and handedness matched with the total mTBI group. In addition to posttraumatic complaints, feelings of anxiety and depression were measured at two weeks using the hospital anxiety and depression scale questionnaire [43].

### Image acquisition and pre-processing

Structural (T1, T2*-gradient echo (T2*-GRE), susceptibility weighted imaging (SWI)) and functional MRI scans were made at approximately four weeks post-injury. Details about image acquisition parameters and fMRI pre-processing were described previously (van der Horn et al., 2016). T2*-GRE and SWI sequences were examined for microbleeds (1-10mm) by an experienced neuroradiologist. Microbleeds were absent in the healthy control group. A total of 158 microbleeds (mean, range: 3, 0–37) were observed in the group of mTBI patients, with zero microbleeds in 72% of the patients. No significant differences in number of microbleeds (*U* = 334, P = 0.88) and percentage of patients with ≥1 microbleeds (χ^2^ = 0.08, P = 0.78) were found between the PTC-present and PTC-absent groups.

### ICA

ICA was performed using the Group ICA of fMRI Toolbox (GIFT) version 3.0a implemented in MATLAB [31]. Similar to Allen and colleagues, prior to ICA decomposition, voxel time series were converted to z-scores to normalize variance across space [35]. A set of 100 components was estimated. Spatial-temporal regression was used for back-reconstruction and ICASSO was repeated 20 times to test component stability [44]. Components were visually characterized as either part of a neural network or as an artefact, based on the expectation that neural networks should exhibit peak activations in grey matter and low spatial overlap with known vascular, ventricular, motion, or susceptibility artifacts. Moreover, power spectra were inspected for dominant low-frequency signal. All components were evaluated by H.J.v.d.H. and E.J.L. separately, and dissimilarities were discussed until consensus was reached.

### Post-processing

Prior to functional connectivity analysis, additional processing steps were applied to the time-courses to remove variance in the data related to white matter (WM) and cerebrospinal fluid (CSF) signal, participants’ motion and scanner drifts [45]. Principal component analysis was applied to the WM and CSF signal and components that explained 95% of the variance were filtered out. Subsequently, linear, quadratic and cubic detrending was performed. Residual effects of motion were corrected by regression with the 6 realignment parameters and their temporal derivatives. Next, temporal band-pass filtering was applied to retain frequencies between 0.008–0.08Hz [45]. Finally, a procedure similar to the one Power and colleagues have used, was carried out to calculate total displacement per scan (i.e. framewise displacement (FD)), and volumes that had a displacement of > 0.5 mm compared to the previous scan were interpolated [35,46]. FD parameters are provided in S1 Appendix. No differences in FD parameters and number of interpolated volumes were found between subgroups.

### Graph analysis

The ICA time courses were correlated using a Pearson’s correlation, and correlations were transformed with a Fisher's Z transformation. We investigated graph measures across a range of thresholds (1–30% strongest connections in the weighted connectivity matrix in steps of 1%). Across this range of thresholds, graph measures were calculated using functions implemented in the Brain Connectivity Toolbox ([24], www.brain-connectivity-toolbox.net). Selection of specific global (computed for the total network) and local (computed for individual nodes) measures was based on previous TBI literature [29,34,47,48] and several comprehensive reviews [22–25]. The following local measures were selected: degree (K_*i*_), strength (S_*i*_), local efficiency (Eloc_*i*_), clustering coefficient (C_*i*_), betweenness centrality (BC_*i*_) and eigenvector centrality (EC_*i*_). Global network measures used in this study were: global efficiency (Eglob), mean local efficiency (Eloc) and mean clustering coefficient (C). A detailed description of these measures is provided by Rubinov and Sporns [24]. Graph measures for every subject at every threshold are provided as S1 Data.

For group comparisons, graph measures were plotted against the threshold range and the area under the curve (AUC) was calculated. These AUC values were compared between patients with mTBI and healthy controls, and between PTC-present patients, PTC-absent patients and healthy controls using permutation testing. Individuals were permuted (retaining original group sizes) and results were recalculated. After 10,000 permutations, significant differences between groups were defined as the outer 0.05 range of the histogram containing these permuted measures. For local measures, multiple comparison correction was performed by calculating the maxima across all nodes per permutation and combining these values in one histogram. Magnitudes of effect were estimated using common language (CL) effect sizes [49]. To gain more insight in the inter-individual variability of complaints in the total patient group, Spearman’s rank correlations were calculated between graph measures and number of complaints. To examine the relationship between network function and emotion regulation, Spearman’s rank correlations were computed between graph measures and anxiety/depression scores in the total group of patients with mTBI. For local measures, false positive correlations were controlled using the false discovery rate (FDR) procedure according to Benjamini and Hochberg (α = 0.05; *m* = number of nodes = 89) [50].

To investigate the influence of structural injury on graph measures, comparisons were made between patients with (n = 15) and without (n = 39) microbleeds on T2*-GRE and SWI. For local measures, multiple comparison correction was performed by combining maxima across all nodes per permutation in one histogram (α = 0.05).

### Module decomposition

Modularity is the extent to which a graph can be divided into modules with a large number of within module connections and a minimal number of between module connections [51]. For fMRI data, such modules have been found to be similar to functional (large-scale) networks [52]. In the current study, it was investigated whether in different groups components belonged to different modules (i.e. large-scale networks). Furthermore, module decomposition was used to aid in the explanation of group differences in local graph measures.

First the optimal threshold for module decomposition had to be determined. Participants’ correlation matrices were binarized at every threshold (1–30%) and these binarized matrices were averaged across all participants (HC and TBI together) [53]. Information theory was applied to compute the entropy (i.e. the amount of distortion) over the averaged matrix for every threshold [54]. The matrix giving the lowest entropy contains the least distortion and therefore has the largest stability across participants. Because entropy depends on the number of elements, a correction was applied by comparing the entropy in the actual matrix to the entropy in randomized matrices. We created 50 randomized matrices per participant, per threshold, preserving the number of nodes and the degree distribution [55]. These were used to construct 500 new average matrices by randomly sampling one of the 50 randomized networks per participant. The entropy was computed and averaged for each of these average random matrices. Subsequently, the optimal threshold was defined as the threshold at which the difference between the entropy in the actual matrix and the entropy in the randomized matrices was maximal.

As input of the partitioning algorithm, averages of the binary matrices were computed per group. Similar to Rubinov and Sporns [56], an initial module partition was created using the algorithm by Blondel *et al*. [57], which attempts to maximize within module connections and minimize between module connections, and this procedure was repeated 500 times. Subsequently, all of these partitions were refined, using a modularity fine-tuning algorithm [58]. Changes that led to an increase in modularity were retained. The fine-tuning algorithm was applied repeatedly until the modularity of the partitioning no longer increased, and the partitioning with the highest modularity was used for further analyses.

To compare the overall module decompositions of HC vs. PTC-present and HC vs. PTC-absent, normalized mutual information (NMI) was used [59], varying from 0 (no mutual information) to 1 (identical node assignments). Statistical differences in module decomposition were analyzed using permutation testing. Participants were randomly divided in groups (retaining original group sizes) and the optimal module decomposition and their NMI were recalculated for each group (repetition: 1000 times). If the actual NMI between groups was smaller than 0.05 of this distribution (i.e. less than 5% of the decomposition of one group could explain the decomposition of the other group), the difference between groups was considered significant.

It was also tested whether specific modules statistically differed between the PTC-present and PTC-absent group and which of them was deviant from HC. To this end, module assignments of both patients groups were categorized using the module decomposition of HC as a reference. Entropy was calculated for both patient groups, with the minimum entropy value indicating that all nodes were in a similar module as the HC’, and the maximum value indicating that they were included in completely different modules. Entropy values of both groups were tested using permutation testing, by randomly changing the patients groups and recalculating the entropy. A difference in entropy of < 0.05 of the distribution was considered significant.

## Results

### Participant characteristics

Fifty-four patients (36 male; mean age 37 ± 15 years) were included in this study. There was a significantly lower percentage of female subjects in the PTC-absent group compared to the PTC-present group (10% and 47%, respectively; χ^2^ = 7.78, P = 0.005). No statistical differences regarding injury severity (Glasgow Coma Scale score and number of patients with posttraumatic amnesia) and injury mechanism (number of patients with mTBI due to traffic-, falls-, sports-, assault-related or other mechanisms) were found between patient subgroups. The PTC-present group reported on average 10 (range: 5–16) complaints, with a mean severity of 13 (range: 5–25). Ninety per cent of the PTC-absent group reported zero complaints. PTC-present patients had higher anxiety (median (interquartile range): 4 (3–7) vs. 2 (0–4.75)), respectively; *U* = 160, P = 0.004) and depression (5 (3–7) vs. 0 (0–1); *U* = 70, P<0.001) scores than PTC-absent patients.

### Group comparisons of graph measures

**Fig 1:**
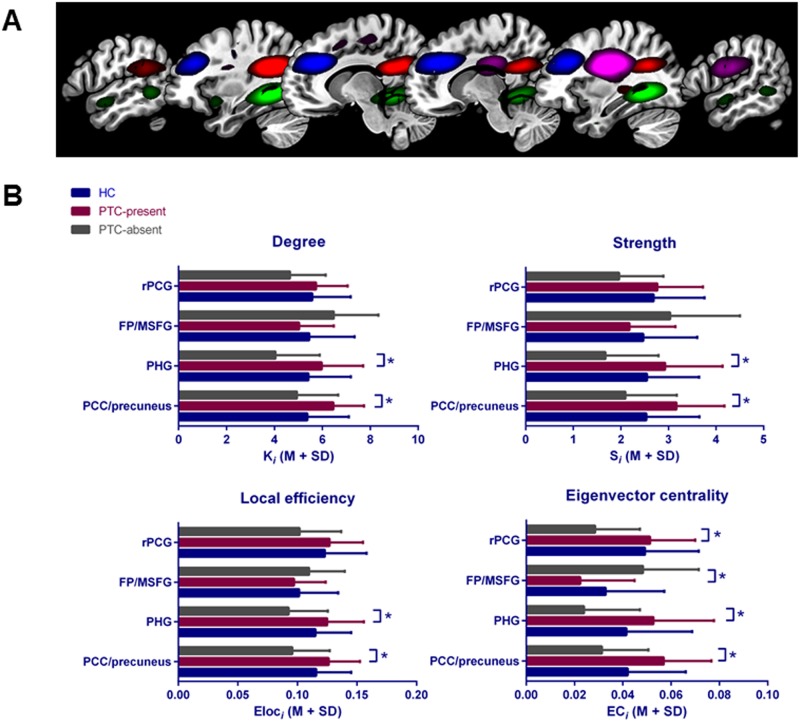
Components that were significantly different between subgroups. A) The bilateral posterior cingulate (PCC)/precuneus is depicted in red; bilateral parahippocampal gyrus (PHG) in green; frontal pole/bilateral middle & sup frontal gyrus (FP/MSFG) in blue and right peri-central gyri (rPCG) in purple; B) Average AUC values for degree (K_*i*_), strength (S_*i*_), local efficiency (Eloc_*i*_) and eigenvector centrality (EC_*i*_) for patients with (PTC-present) and without (PTC-absent) complaints and healthy controls (HC). Asterisks indicate significance p<0.05 after correction for multiple comparisons.

After ICA, 89 components were identified as neural networks and included in network analyses. Permutation testing did not show differences between HC and the total group of patients with mTBI for any of the calculated graph measures. Regarding patient subgroups, PTC-present patients had higher values compared to PTC-absent patients on the following local graph measures: degree of the bilateral posterior cingulate cortex (PCC)/precuneus (P<0.0009; CL = 0.76) and the bilateral parahippocampal gyrus (PHG) (P<0.0006; CL = 0.78), strength of the bilateral PCC/precuneus (P<0.0009; CL = 0.77) and bilateral PHG (P<0.0009; CL = 0.78), local efficiency of the bilateral PCC/precuneus (P<0.0004; CL = 0.77) and bilateral PHG (P<0.0004; CL = 0.76), and eigenvector centrality of the bilateral PCC/precuneus (P<0.0001; CL = 0.82), bilateral PHG (P<0.0003; CL = 0.8) and right peri-central gyri (PCG) (P <0.0001; CL = 0.81) (Fig 1). In contrast, PTC-present patients had lower eigenvector centrality of the frontal pole (FP)/bilateral middle & sup frontal gyrus (MSFG) (P <0.0003; CL = 0.21) compared to the PTC-absent group. For global efficiency, mean local efficiency and mean clustering coefficient, no significant differences were found between patient subgroups.

No significant differences in graph measures were found between patients with and without microbleeds on T2*-GRE and SWI.

### Graph measures related to the number of complaints

Table 1 lists graph measures of the components that were significantly correlated with number of complaints in the patient group. Regarding the components that were different between PTC-present and PTC-absent patients, graph measures of the bilateral PCC/precuneus and PHG were positively correlated with number of complaints, while graph measures of FP/MSFG were negatively related to number of complaints.

**Table 1:** Spearman’s rank correlations between graph measures and number of complaints in the patient group (significant at FDR < 0.05).

*component*	K*i*	S*i*	EC*i*
**Bilateral PCC/precuneus**	0.43		0.52
(peak MNI: x = -27, y = -45, z = 24)			
**Bilateral parahippocampal gyrus**	0.51	0.50	0.51
(peak MNI: x = -24, y = -42, z = -6)			
**Left inf/sup parietal lobe**	-0.41		-0.40
(peak MNI: x = -39, y = -39, z = 42)			
**Right inf/sup parietal lobe**	-0.43		-0.44
(peak MNI: x = 45, y = -36, z = 42)			
**Right peri-central gyri**			0.46
(peak MNI: x = 27, y = -18, z = 36)			
**Frontal pole/ bilateral middle & sup frontal gyrus**	-0.41		-0.43
(peak MNI: x = 27, y = 36, z = 30)			
**ACC/middle & sup frontal gyrus**			0.43
(peak MNI: x = 6, y = 33, z = 18)			
**Middle/posterior cingulate gyrus**			-0.38
(peak MNI: x = 0, y = -3, z = 36)			
**Bilateral frontal operculum/insula**			0.42
(peak MNI: -24–3 18)			
**Right middle frontal/precentral gyrus**			-0.39
(peak MNI: x = 27, y = -6, z = 39)			

*Abbreviations*: ACC = anterior cingulate cortex; EC*i* = node eigenvector centrality; FDR = false discovery rate; inf = inferior; K*i* = node degree; MNI = Montreal Neurological Institute; PCC = posterior cingulate cortex; PTC = posttraumatic complaints; S*i* = node strength; sup = superior.

### Graph measures related to anxiety and depression

Table 2 lists graph measures of the components that were significantly correlated with depression scores in the total group of patients with mTBI. Whereas graph measures of posterior midline areas were positively correlated with depression scores, graph measures of lateral frontoparietal areas were negatively correlated with depression scores. No significant correlations were observed between local graph measures and anxiety scores. Global network measures showed no significant correlations with either anxiety or depression scores.

**Table 2:** Spearman’s rank correlations between graph measures and depression scores in the patient group (significant at FDR < 0.05).

*component*	K*i*	S*i*	EC*i*
**Bilateral PCC/precuneus**	0.40		0.47
(peak MNI: x = -27, y = -45, z = 24)			
**Bilateral parahippocampal gyrus**	0.49	0.47	0.46
(peak MNI: x = -24, y = -42, z = -6)			
**Left inf/sup parietal lobe**	-0.45		-0.49
(peak MNI: x = -39, y = -39, z = 42)			
**Right inf/sup parietal lobe**	-0.54		-0.53
(peak MNI: x = 45, y = -36, z = 42)			
**Bilateral inf/sup parietal lobe**			-0.43
(peak MNI: x = -21, y = -30, z = 45)			
**Frontal pole/bilateral middle & sup frontal gyrus**	-0.44		-0.47
(peak MNI: x = 27, y = 36, z = 30)			
**Bilateral middle frontal gyrus**			-0.48
(peak MNI: x = -27, y = 63, z = 24)			
**Bilateral lingual gyrus**	-0.44		
(peak MNI: x = 12, y = -90, z = -6)			
**Bilateral calcarine sulcus**	0.41		
(peak MNI: x = -18, y = -66, z = 6)			

*Abbreviations*: EC*i* = node eigenvector centrality; FDR = false discovery rate; inf = inferior; K*i* = node degree; MNI = Montreal Neurological Institute; PCC = posterior cingulate cortex; S*i* = node strength; sup = superior.

### Module decomposition

**Fig 2:**
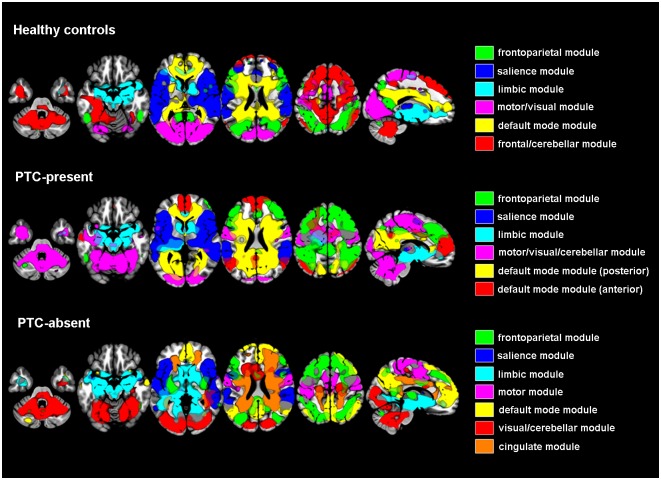
Module decomposition per subgroup. Overlays were constructed per subgroup using one-sample *t*-tests in SPM12, and *T*-thresholds were adjusted separately for every overlay to ensure optimal display.

The optimal threshold for connections to retain in the correlation matrix was 1.85%. Results of module decomposition are shown in Fig 2. Several differences between groups can be noticed. Most strikingly, while the HC and PTC-present groups both had six modules, estimation resulted in seven modules for the PTC-absent group. In this group, there appeared to be a separate module consisting mainly of the cingulate areas. In the HC and PTC-absent group, most of these areas were incorporated in the default mode module(s). Despite visual dissimilarities and the fact that PTC-absent patients had an extra module, there were no statistically significant group differences in module decomposition.

To aid in the interpretation of our graph results, we assessed which modules contained the components that showed significant correlations with posttraumatic complaints and/or depression scores. Module assignment for these components is listed per subgroup in Table 3. Noticeably, in the PTC-present and HC groups, the bilateral PCC/precuneus and PHG components were both assigned to the default mode module, while in the PTC-absent group the bilateral PCC/precuneus and PHG were assigned to the cingulate and limbic module, respectively. The FP/ bilateral MSFG was included in the frontoparietal, salience and cingulate module for the PTC-present, HC and PTC-absent group, respectively.

**Table 3:** Module assignment for each component that was significantly related to posttraumatic complaints and/or depression scores.

*Component*	PTC-present	PTC-absent	HC
Bilateral PCC/precuneus	Default mode	Cingulate	Default mode
Bilateral parahippocampal gyrus	Default mode	Limbic	Default mode
Left inf/sup parietal lobe	Frontoparietal	Frontoparietal	Frontoparietal
Right inf/sup parietal lobe	Frontoparietal	Frontoparietal	Frontoparietal
Bilateral inf/sup parietal lobe	Frontoparietal	Cingulate	Frontal/cerebellar
Right peri-central gyri	Default mode	Cingulate	Default mode
Frontal pole/bilateral middle & sup frontal gyrus	Frontoparietal	Cingulate	Salience
ACC/ bilateral middle & sup frontal gyrus	Default mode	Cingulate	Default mode
Middle/posterior cingulate gyrus	Frontoparietal	Frontoparietal	Salience
Bilateral frontal operculum/insula	Default mode	Cingulate	Default mode
Right middle frontal/precentral gyrus	Frontoparietal	Frontoparietal	Frontal/cerebellar
Bilateral middle frontal gyrus	Frontoparietal	Frontoparietal	Frontal/cerebellar
Bilateral lingual gyrus	Motor/visual/cerebellar	Visual/cerebellar	Motor/visual
Bilateral calcarine sulcus	Default mode	Limbic	Motor/visual

*Abbreviations*: ACC = anterior cingulate cortex; HC = healthy controls; inf = inferior; PTC = posttraumatic complaints; PCC = posterior cingulate cortex; sup = superior.

## Discussion

In this study, graph analysis of functional brain networks was performed in patients with uncomplicated mTBI in the sub-acute phase after injury, aimed to improve our understanding of the presence of posttraumatic complaints related to anxiety and depression after mTBI. A large patient sample was included, which provided us with sufficient power to detect possible group differences. None of the network measures differed between patients with mTBI and healthy controls, but in patient subgroups various differences were found in local network measures of prefrontal and parietal midline and parahippocampal areas. In patients with mTBI, associations were found between local network measures and depression scores, but not between any of the network measures and anxiety. Module decomposition was similar for all study groups, although patients without complaints showed one extra module compared to patients with complaints and healthy controls, which was composed of the cingulate areas.

Global network measures did not differ between patients with and without complaints, which may be consistent with the fact that cognitive functioning is unimpaired in most of the cases of mTBI [5–7]. Regarding local measures, however, higher values of degree, strength, local efficiency and eigenvector centrality of the bilateral PCC/precuneus and bilateral PHG were found in patients with complaints compared to patients without complaints. Within the group of patients with mTBI, higher degree, strength and eigenvector centrality of these components were also associated with a higher number of complaints. In partial agreement with our results, a study by Messe *et al*. demonstrated higher graph measures of the left PHG in mTBI patients with complaints in the sub-acute phase compared to healthy controls [29]. Furthermore, studies using non-graph analyses have reported higher functional connectivity within the posterior midline and parahippocampal areas in patients with chronic mild to severe TBI compared to healthy controls [60,61].

Over the past few years, evidence has accumulated that network dynamics play a crucial role in the development of posttraumatic complaints after mTBI [12–14]. Network dynamics strongly rely on hub nodes, and these nodes can be affected by TBI, especially moderate-to-severe TBI [32]. The PCC/precuneus and PHG are regarded as hub nodes in the human brain [62–64]. They are key areas of the DMN, but are also incorporated in executive networks during externally focused cognition [62,63,65,66]. The PHG can be considered part of the medial temporal lobe subsystem of the DMN that is involved in autobiographical memory [64,67]. We have shown that in patients with complaints the bilateral PCC/precuneus and PHG were both included in the (posterior) default mode module. In patients without complaints, however, the bilateral PCC/precuneus was incorporated in the cingulate module and the bilateral PHG was included in the limbic module. It could be hypothesized that stronger connectivity of the PCC and PHG within the DMN in patients with complaints is associated with ongoing negative self-referential mental processes, such as worrying about subjective cognitive problems, mood problems, negative illness beliefs or expectations about future situations [15,68]. Furthermore, our findings might be related to posttraumatic stress, because posttraumatic complaints strongly overlap with symptoms of the hyperarousal dimension of posttraumatic stress disorder [69]. In veterans with mTBI, associations were found between re-experiencing symptoms and weaker functional connectivity of a functional network including basal ganglia, prefrontal cortex, insula and posterior cingulate cortex [30]. It could be possible that because of stronger connectivity within the limbic and cingulate modules and weaker connectivity within the default mode module, patients without complaints may be more resilient to stress and less prone to developing complaints. As the default mode network, executive network and salience network converge within the midline (cingulate areas), it could also be postulated that the extra cingulate module in patients without complaints is associated with more balanced switching between internally and externally focused mental processes leading to more optimal cognitive and emotional processing and stress regulation compared to patients with complaints [18,20,21,63,70]. Since this cannot be directly deduced form our data, future studies are required to confirm our theories.

Eigenvector centrality of the FP/bilateral MSFG was lower in patients with complaints compared to those without complaints, which is in contrast to the posterior regions. To put it differently, nodes neighboring the FP/ bilateral MSFG were less likely to be hubs in these patients, indicating weaker connections throughout the prefrontal cortex. These findings complement previous non-graph studies on mTBI that have shown that lower functional connectivity of prefrontal areas was associated with a higher number of complaints and higher anxiety and depression scores [29,71,72]. The prefrontal cortex acts as a relay station in the interaction between networks involved in cognitive and emotional functioning [15,18,19,70]. Therefore, our results may point towards an association between posttraumatic complaints and disturbances in network dynamics that may (partly) arise from prefrontal dysfunction.

Regarding the presence or absence of posttraumatic complaints, it has to be noted that based on the literature a relatively low percentage of patients with mTBI report to have no complaints at all after (civilian) mTBI [41,42]. Moreover, even healthy controls have been found to report on average more than one complaint [42]. To our knowledge, the group of patients without any complaints has not received much attention so far, especially in functional neuroimaging studies, but is very interesting with regard to studying mechanisms that are related to successful recovery. It is therefore a unique feature of our study that we succeeded in including a relatively large group of patients without complaints, in addition to a group with complaints.

Interestingly, graph measures of posterior components that are associated with the DMN were positively correlated with depression scores, while measures of frontal and parietal components that are generally associated with executive networks were negatively correlated with depression scores in the total group of patients with mTBI. In concordance with our results, increased functional connectivity of the default mode network has been consistently observed in major depressive disorder [68,73]. Furthermore, the executive networks are thought to form a key regulatory system for promoting and maintaining mental health [21]. Prefrontal areas, such as the middle and superior frontal gyrus and the anterior cingulate cortex, are crucial area in this process, because of their role in emotion regulation [20]. Our current findings extend on our previous work on the same patient sample that demonstrated that prefrontal brain networks are important for emotion regulation after mTBI [13]. It has to be realized that causal inference based on our observed correlations is not possible, since higher values of graph measures might lead to lower depression scores, but the reverse is also plausible. Nevertheless, our findings suggest that intervention therapies targeted at executive functioning and attention may also improve emotion regulation in patients with mTBI [74,75].

Previous research has shown lower local and global graph measures of functional networks in patients with mild-to-severe TBI compared to healthy controls, and these measures were associated with traumatic axonal injury [34]. In our study, we found no differences in graph measures between patients and healthy controls, although we investigated a patient group at the milder end of the TBI spectrum. Furthermore, it might seem counterintuitive that patients with complaints did not differ from healthy controls, because these patients are clinically most affected. These findings cast doubts on the causative role of mTBI itself in functional network connectivity differences between patients with and without complaints. These doubts are strengthened by the fact that graph measures were similar for patients with and without micro-hemorrhagic lesions. Interestingly, recent research demonstrated that high pre-injury somatization scores predicted longer symptom duration after sports-related concussion [76]. Furthermore, variations in graph measures have been associated with personality characteristics, such as neuroticism, in healthy subjects [77]. Therefore, it is tempting to hypothesize that also graph analysis findings in mTBI are not injury-related, but associated with pre-injury personality characteristics that predispose to developing complaints after a stressful event, such as a mTBI [15,78].

In the current study, high order ICA model was combined with graph analysis [33,35,37] aimed at discerning subtle changes in large-scale network function that possibly remained hidden in previous analyses [13]. Group ICA was used to define network nodes because this data-driven method has been shown to adequately capture inter-individual differences, and may result in more accurate *functional* components for the dataset that is being investigated [33,79]. However, the ‘best’ method for node definition is unknown, and various other effective parcellation methods are available [23,33,52,80]. Although volume of between-group differences has shown to be optimal at a model order of 70 to 100, high order models have its disadvantages as the exact number of selected components is rather arbitrary, and power is possibly reduced considering the large number of tests that are performed and have to be statistically corrected, especially regarding local measures [37]. The selection of graph measures of the current fMRI study was based on graph measures that were used in previous TBI studies [29,34,47,48], and aimed to obtain an impression of network Integration, Segregation and Influence [25]. However, it is still largely unclear which measures are most informatory for investigating (m)TBI. Based on the results of our study, it may be worthwhile to use eigenvector centrality in future research on mTBI. Lastly, computing modules for different study subgroups provided an interesting perspective on large-scale network function after mTBI. Still, caution is warranted in interpreting our module decomposition results, because no statistical group differences were found.

A limitation of our study is the lack of data about complaints, anxiety and depression in the healthy control group, because complaints are to some extent also reported by healthy controls [42]. However, in the patient groups we corrected for pre-injury levels of functioning by subtracting their pre-injury complaint scores from post-injury scores (i.e. scores reflect complaints that developed post-injury). Furthermore, there was an interval of 1–2 weeks between filling out the two weeks questionnaire and the appointment for an MRI scan. Therefore, it is possible that at time of scanning, patients would have reported fewer complaints. However, considering the short interval and the high average number and severity of complaints at two weeks, we deem it unlikely that complaints would be greatly decreased at time of scanning. Lastly, we did not administer neuropsychological tests at time of scanning. However, it is known from previous studies that cognitive deficits are often absent in the sub-acute phase after mTBI [5–7].

## Conclusions

In this study, a novel approach, consisting of high order ICA followed by graph analysis, was used to investigate functional brain networks in relation to complaints, anxiety and depression after mTBI. Interestingly, all network measures were similar for patients with mTBI and healthy controls, which might suggest that the influence of the injury itself in network function after mTBI is not that strong. Regarding patient subgroups, higher local graph measures were found in patients with complaints compared to patients without complaints, especially in default mode network related areas in the proximity of the posterior midline. In addition, higher values of these components were related to mood disturbances in patients with mTBI, while the opposite was true for components of the executive networks. It could be hypothesized that targeting mood problems after mTBI, with therapies focused on executive functioning, may lead to a reduction of complaints. More studies are required to further elucidate the complex alterations in functional networks after mTBI, with an emphasis on personality characteristics and emotion regulation.

## Supporting Information

S1 Appendix Framewise Displacement parameters. For every subject minimum and maximum framewise displacement (FD), number of volumes with > 0.5 mm FD, and % volumes that had a displacement of > 0.5 mm are listed.
(XLS)

S1 Data Graph Measures. Graph measures for every subject at every threshold.
(ZIP)
